# Systematic Differences Between Perceptually Relevant Image Statistics of Brain MRI and Natural Images

**DOI:** 10.3389/fninf.2019.00046

**Published:** 2019-06-25

**Authors:** Yueyang Xu, Ashish Raj, Jonathan D. Victor

**Affiliations:** ^1^Department of Electrical Engineering, Stanford University, Stanford, CA, United States; ^2^Department of Radiology & Biomedical Imaging, University of California, San Francisco, San Francisco, CA, United States; ^3^Department of Neurology and Feil Family Brain and Mind Research Institute, Weill Cornell Medical College, New York, NY, United States

**Keywords:** magnetic resonance imaging, brain, image statistics, human vision, efficient coding

## Abstract

It is well-known that the human visual system is adapted to the statistical structure of natural scenes. Yet there are important classes of images – for example, medical images – that are not natural scenes, and therefore, that are expected to have statistical properties that deviate from the class of images that shaped the evolution and development of human vision. Here, focusing on structural brain MRI images, we quantify and characterize these deviations in terms of a set of local image statistics to which human visual sensitivity has been well-characterized, and that has previously been used for natural image analysis. We analyzed MRI images in multiple databases including T1-weighted and FLAIR sequence types, and simulated MRI images based on a published image simulation procedure for T1 images, which we also modified to generate FLAIR images. We first computed the power spectra of MRI images; spectral slopes were in the range −2.6 to −3.1 for T1 sequences, and −2.2 to −2.7 for FLAIR sequences. Analysis of local image statistics was then carried out on whitened images. For all of the databases as well as for the simulated images, we found that the three-point correlations contributed substantially to the differences between the “texture” of randomly selected ROIs. The informative nature of three-point correlations for brain MRI was greater than for natural images, and also disproportionate to human visual sensitivity. As this finding was consistent across databases, it is likely to result from brain geometry at the scale of brain MRI resolution, rather than characteristics of specific imaging and reconstruction methods.

## Introduction

Development of image processing systems and algorithms is often guided by the strategies used by the human visual system. A main motivation for this approach is that drawing inferences from images is a complex and ill-posed problem, but one that the human visual system, as a result of evolutionary and developmental forces, has become reasonably effective at solving.

What is unclear, however, is the level of detail at which human vision should be used as a source to guide machine analysis of medical images: not only does human vision operate under different constraints than machine vision, but also, it is matched to the statistical properties of images that result from projections of the natural environment onto the retina. This matching is at a surprising degree of detail, encompassing not only the well-recognized stage of redundancy reduction by removal of global correlations (Atick and Redlich, 1992; Rucci and Victor, 2015), but also the allocation of resources to the analysis of local image statistics in a way that is closely matched to their informative value (Hermundstad et al., 2014). While the computational strategies used by human vision are sufficiently robust and general to enable perceptual judgments about images that are highly non-natural – for example, modern art – there is ample evidence that these strategies reflect a specific allocation of computational resources: for example, some kinds of local correlations are readily perceptible, while others, which are of comparable mathematical complexity, escape our notice (Tkacik et al., 2010).

Therefore, understanding the extent to which a particular image class shares the statistical properties of natural images is likely to be helpful in translating the lessons learned by the human visual system into specific applications. Here, we focus on this comparison for structural brain MRI.

Because the human visual system is matched to natural images both in terms of their global and local statistical properties, we consider these aspects of brain MRI images independently. With regard to characterization of the global statistical properties, we use the power spectrum (McVeigh et al., 1985), as it is a principled approach that provides a full and concise characterization of the correlation structure of a stationary Gaussian image ensemble. However, it is well-recognized that medical images are non-Gaussian (Burgess et al., 2001; Theocharakis et al., 2009; Abbey et al., 2012) and, moreover, that these non-Gaussian aspects are functionally important, as they affect the ability to detect abnormalities (Burgess et al., 2001).

In contrast to approaches for analysis of the Gaussian aspects of an image set – for which the power spectrum is pre-eminent – there are many options for quantification of non-Gaussian characteristics. The basic reason for this diversity is that Gaussianity is a strong assumption, so it can be tested in many ways. For example, in a stationary Gaussian image ensemble, the output of any linear filter will have a Gaussian distribution, so any deviation from this distribution – for any filter shape – is a candidate measure of non-Gaussian behavior. Thus, deviations from Gaussian behavior can be captured by direct characterization of the moments of such distributions (Theocharakis et al., 2009), or indirect characterizations, e.g., via the entropy of the best-fitting Laplacian (Abbey et al., 2012).

While these approaches are effective ways of capturing non-Gaussian aspects of medical images, here, we use another strategy for assessment of non-Gaussian aspects of an image set, which, along with entropic methods (Abbey et al., 2012), is motivated by relevance to human vision (Victor and Conte, 2012; Hermundstad et al., 2014). However, in contrast to the strategy used previously (Abbey et al., 2012) that takes the viewpoint of physiology, here we take the viewpoint of perception. That is, rather than measure non-Gaussian aspects of an image set by characterizing the outputs of filters designed to model the receptive fields of cortical neurons (i.e., Gabor functions Marcelja, 1980), we analyze images according to statistics motivated by analyses of human perceptual sensitivity (Victor and Conte, 2012; Victor et al., 2013, 2015; Hermundstad et al., 2014). The analysis is carried out on whitened images, so it is independent of any overall differences in the power spectrum, and allows for a direct comparison to a previous analysis of natural images (Hermundstad et al., 2014).

The motivation for taking a perceptual approach rather than a physiological one is that current models of cortical visual processing at the neuronal level are quite incomplete: they provide a reasonable account for responses to simple stimuli such as sinusoidal gratings and noise (Rust and Movshon, 2005), but they account for less than half of the variance for responses to more naturalistic stimuli (Willmore et al., 2010), and they especially fail when high-order correlations are present (Joukes et al., 2017). So, although it is anticipated that visual perception is ultimately explainable on the basis of neural function, at present there is a substantial gap between models of visual processing based on neurons as filters, and perception.

In brief, our approach characterizes the non-Gaussian aspects of an image in terms of the intensity patterns that occur within a 2 × 2 region of checks (Tkacik et al., 2010; Victor and Conte, 2012; Hermundstad et al., 2014; Victor et al., 2015), and organizes this characterization into measures of two-, three-, and four-point correlations. We find commonalities of these fingerprints of non-Gaussian behavior across T1-weighted and FLAIR sequences – specifically, that three-point correlations are of greater importance in brain MRI images than in natural images, and therefore disproportionately large compared to human visual sensitivity. We speculate on the basis of these findings, and their possible relevance for the interpretation of brain MRI.

## Materials and Methods

### Databases and Image Selection

Analyses were carried out on de-identified human brain MRI images obtained from three databases and on simulated MRI images computed by BrainWeb (Coscosco et al., 1996; Kwan et al., 1996, 1999; Collins et al., 1998). The human MRI databases were: the Open Access Series of Imaging Studies (OASIS) (Marcus et al., 2007) the Alzheimer Disease Neuroimaging Initiative (ADNI) (Mueller et al., 2005) and a dataset from healthy volunteers and individuals diagnosed with a variety of neuroinflammatory diseases (mostly multiple sclerosis) collected in the Translational Neuroradiology Section of National Institute of Neurologic Disorders and Stroke (NINDS), here designated the TNS dataset. The TNS dataset was provided by Daniel S. Reich at the NINDS.

All images were analyzed as sagittal slices, with a voxel size of 1.0 mm × 1.0 mm × 1.0 mm for the ADNI and TNS databases, and 1.0 mm × 1.0 mm × 1.25 mm for the OASIS database. For the OASIS and ADNI databases, images were obtained using T1-weighted sequences (Mueller et al., 2005; Marcus et al., 2007). For the TNS database, images were obtained with a T2-weighted FLAIR sequence.

**FIGURE 1:**
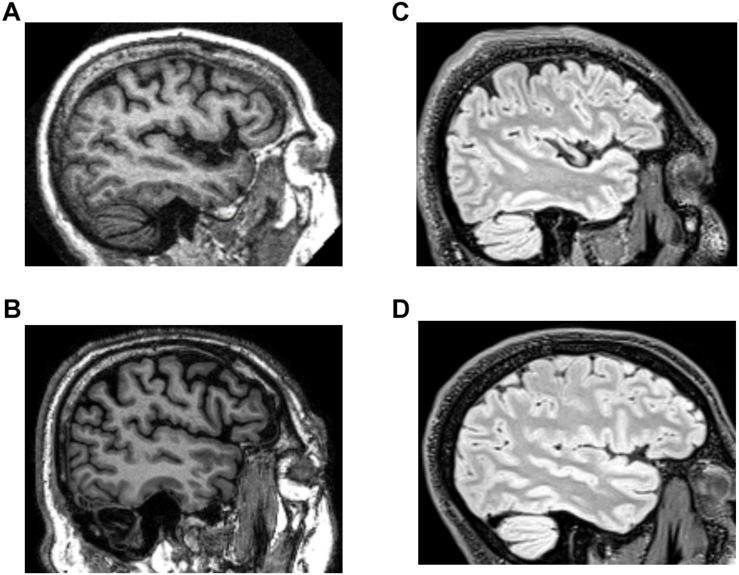
Example images from the structural brain MRI databases. **(A,B)** T1 images from the OASIS and ADNI databases. **(C,D)** T2-weighted FLAIR images from the TNS database (healthy subject, **C**; patient subject, **D**).

Example images from these databases are shown in Figure 1. T1-weighted and T2-weighted FLAIR images differ in the aspects of brain tissue that determine image intensity. Briefly, in a T1-weighted MRI image, intensity depends predominantly on the spin-lattice relaxation time of tissue; fat and white matter appear bright. In a T2-weighted MRI image, intensity depends predominantly on the spin-spin relaxation time of tissue; normal white matter appears dark, while CSF and many brain lesions appear bright. In T2-weighted FLAIR, signal from CSF is suppressed.

Characteristics of the databases are summarized in Table 1. The OASIS database, 380 image datasets, consists of healthy subjects and patients with a variety of diagnoses, age range 18–96 years. The ADNI database, 119 image datasets, consists of patients diagnosed with Alzheimer’s disease, age range 55–90 years. The TNS database had two subsets: one with images from 39 healthy subjects and one with images from 255 patients suspected of having neuroinflammatory diseases, such as multiple sclerosis; combined age range was 18–83 years. All databases included male and female subjects, and all the images in each database were analyzed. As detailed below, this yielded a total of 67,908 regions of interest (ROIs) from 793 brain volumes (Table 1). Note that the analyzed sets of images differed not only in the medical status of the individuals who were imaged, and but also in age range. Additionally, there were likely subtle differences in imaging and reconstruction methods, subject selection criteria, movement artifact, and other factors. Our intent was not to identify statistical features of images that were specific to any of these factors, but rather, to identify statistical features shared by all the datasets, and thus, were common to brain MRI as a whole.

**TABLE 1:** Characteristics of the MRI databases used in this study.

								**Field**	**Number of**	**Number of**	**Mean ROIs**
**Database**	**Age**				**Gender**	**Diagnosis**	**Sequence**	**strength**	**brains**	**ROIs**	**per brains**
	**Min**	**Max**	**Mean**	**SD**							
OASIS	18	96			M+F	Various	T1	1.5T	380	28951	76.2
ADNI	55	90			M+F	AD	T1	3T	119	9500	79.8
TNS healthy	20	64	36.3	12.5	M+F	Healthy	FLAIR	3T	39	4289	110.0
TNS patient	18	83	48.2	12.5	M+F	MS	FLAIR	3T	255	25168	98.7
**Total**									**793**	**67908**	**85.6**

*AD, Alzheimer’s Disease; MS, multiple sclerosis.*

To help distinguish between the influences of brain structure and the influences of acquisition noise and reconstruction, we also analyzed simulated T1-weighted and FLAIR images. Simulated T1 weighted MRI images were calculated using the BrainWeb simulator (Kwan et al., 1996, 1999; Collins et al., 1998). We used the system defaults, namely, a voxel size of 1.0 mm × 1.0 mm × 1.0 mm and spoiled FLASH with TR = 18 ms, TE = 10 ms, flip angle 30°. FLAIR MRI images with CSF suppression were not supported by the online BrainWeb simulator. To obtain these images, we modified the simulator code to use a T1 relaxation time of 4500 ms for CSF instead of the default value of 2569 ms, and a T2 relaxation time of 2300 ms for CSF rather than the default value of 329 (Condon et al., 1987) and simulated an inversion-recovery sequence with TR = 11000 ms, TE = 140 ms, TI = 4600 ms. These modifications required recompiling the simulator code locally, using Ubuntu 16.04.2. For both types of images, noise was simulated as additive Gaussian white noise, and its standard deviation was specified as a fraction of the most intense tissue value (0, 3, 5, or 7%).

### Processing Pipeline

For each database, the processing pipeline consisted of three stages: (i) extraction of regions of interest (ROIs), (ii) computation of the power spectra within each database and whitening of the ROIs via these spectra, and (iii) computation of local image statistics from whitened, binarized ROIs. The second and third stages were identical to those used by Hermundstad et al. (2014) in the analysis of natural images, and will be summarized here for the reader’s convenience.

**FIGURE 2:**
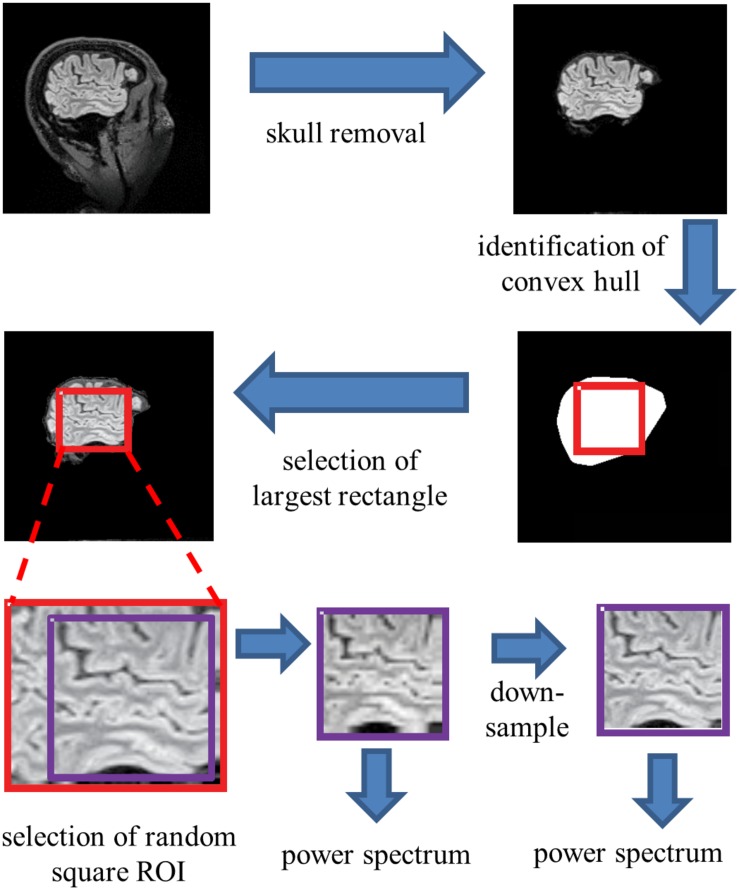
Processing pipeline for spectral analysis. Images of each sagittal plane were processed by (1) removal of the skull, (2) identification of the convex hull of the resulting image, (3) selection of the largest rectangle, and (4) selection of a random 64***×***64 pixel square ROI within this convex hull. The power spectrum was then computed from the mean of the squared amplitudes of the Fourier components obtained from these ROIs. Optionally, the image was then downsampled by averaging within 2***×***2 blocks prior to Fourier transformation.

To extract individual ROIs that were fully contained within brain parenchyma in an automated, unbiased fashion, we proceeded as follows (Figure 2). For each sagittal section, the skull was stripped using Freesurfer’s “watershed.” Then, using Matlab’s bwconvhull, the convex hull was determined, and the largest rectangle that fit inside this region was then found. Next, we randomly selected one 64 × 64 ROI from inside each rectangle (slices whose convex hulls were too small to contain a 64 × 64 rectangle were discarded). To probe the possible effects of high-frequency artifacts in the reconstruction, a parallel analysis was carried out after downsampling the 64 × 64 ROIs to 32 × 32, by averaging the intensities in 2 × 2 blocks. We present data from both the full-resolution and the down-sampled analyses in parallel.

To compute spatial power spectra, we applied Matlab’s fft2 in each ROI, and averaged the squared magnitudes of the Fourier components across all slices from each database. Spectra were computed both with and without twofold padding. Padding had minimal effect and was not used in the figures below. For simplicity, we did not use windowing; the use of windows (e.g., a multitaper approach Mitra and Pesaran, 1999) would have little effect because the spectra are broadband.

The shape of the power spectra was summarized by power-law fits. These were obtained by regressing the logarithm of the spectral densities against the logarithm of spatial frequency (using Matlab’s regress), over the frequency range from 2 cycles per ROI (0.031 cycles/mm) to 10% below the Nyquist frequency (0.45 cycles/mm for the full-resolution images, 0.225 cycles/mm for the downsampled images). The zero-frequency power, which depends on the arbitrary choice of the image intensity values, has no influence on this analysis as it only considered spatial frequencies beginning at 2 cycles per ROI.

The final stage of the pipeline is the computation of local image statistics. These statistics capture the local correlations that are present in small neighborhoods after whitening via the empirical spectra (not the power-law fits), and therefore reflect non-Gaussian aspects of the images. We focused on characterizing the distribution of colorings of 2 × 2 blocks of binarized pixels. As described below, these distributions can be described by a discrete set of nine parameters. As mentioned above, the corresponding image statistics are known to be perceptually relevant: their salience to human observers has been well-characterized individually and in combination (Hermundstad et al., 2014; Victor et al., 2015).

**FIGURE 3:**
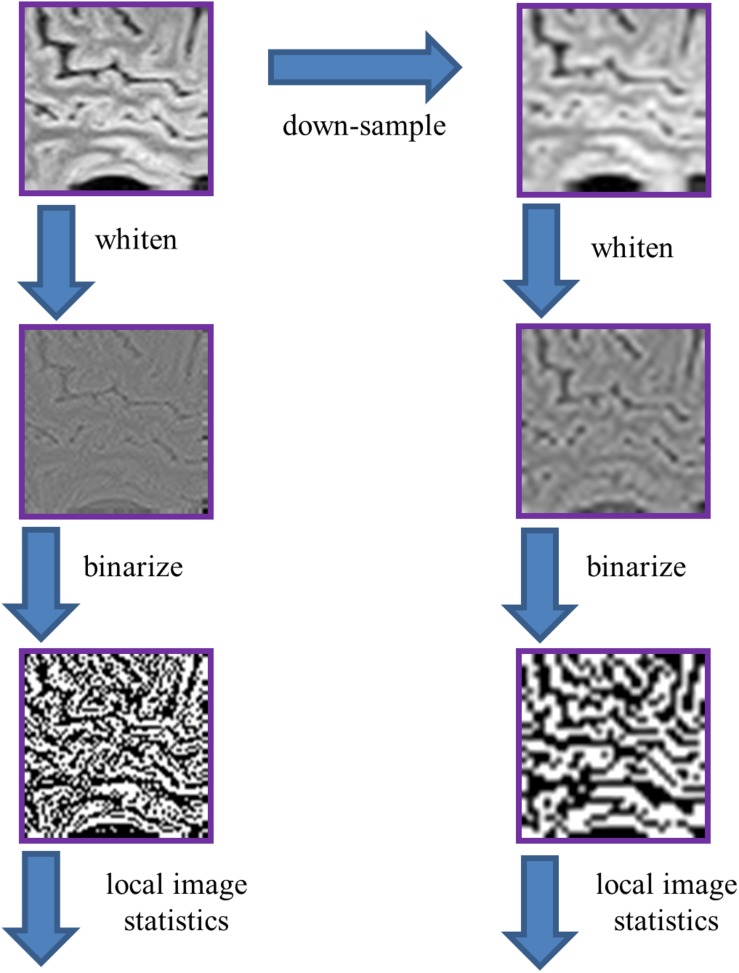
Processing pipeline for analysis of local image statistics. The ROIs extracted for spectral analysis (Figure 2) were whitened by a linear filter whose amplitude is given by the square root of the power spectrum for that dataset. The resulting images were then binarized at the median value of the whitened images. Local image statistics were determined by tabulating the configurations of black and white checks within 2 × 2 neighborhoods.

To extract these statistics (Figure 3), we used the procedure developed in Hermundstad et al. (2014) (their Figure 2), with parameters *R* (ROI size) and *N* (downsampling) of (*R*,*N*) = (64,1) and (*R*,*N*) = (32,2). The larger (*R*,*N*) parameter values used by Hermundstad et al. (2014) for natural images (*R*× *N*>64) could not be used, because the limited number of voxels per plane in the MRI images would often not allow for randomized placement of these patches within brain. As in Hermundstad et al. (2014), we whitened these ROIs by filtering them in the frequency domain, attenuating each Fourier component by the inverse square-root of the power spectrum (across the entire frequency range), computed over all the ROIs within the image’s database. Then, these images were binarized at the median point, where the median was determined from all of the ROIs drawn from the same brain. Note again that the zero-frequency power does not influence the results: although it scales the intensity range via the whitening process, the effect of scaling is removed once images are binarized at their median. The binarized images were then parceled into 2 × 2 blocks, after removing a one-pixel border to avoid edge artifacts due to the whitening operation.

Continuing as in Hermundstad et al. (2014), we parameterized the distribution of colorings of these 2 × 2 blocks within an ROI as follows. Since each of the pixels in a block were either black or white, there are 16 ($2^{2\times 2}$) different ways that a 2 × 2 block can be colored. However, fewer than 16 degrees of freedom are required to describe them. These constraints arise because (a) the 16 probabilities add up to 1, and (b) the left two pixels of one block are also the right two pixels of the next (and similarly, for top and bottom). To take these constraints into account, we used the parameter set of Victor and Conte (2012), which linearly transforms the 16 interdependent raw block probabilities into 10 independent statistics.

**FIGURE 4:**
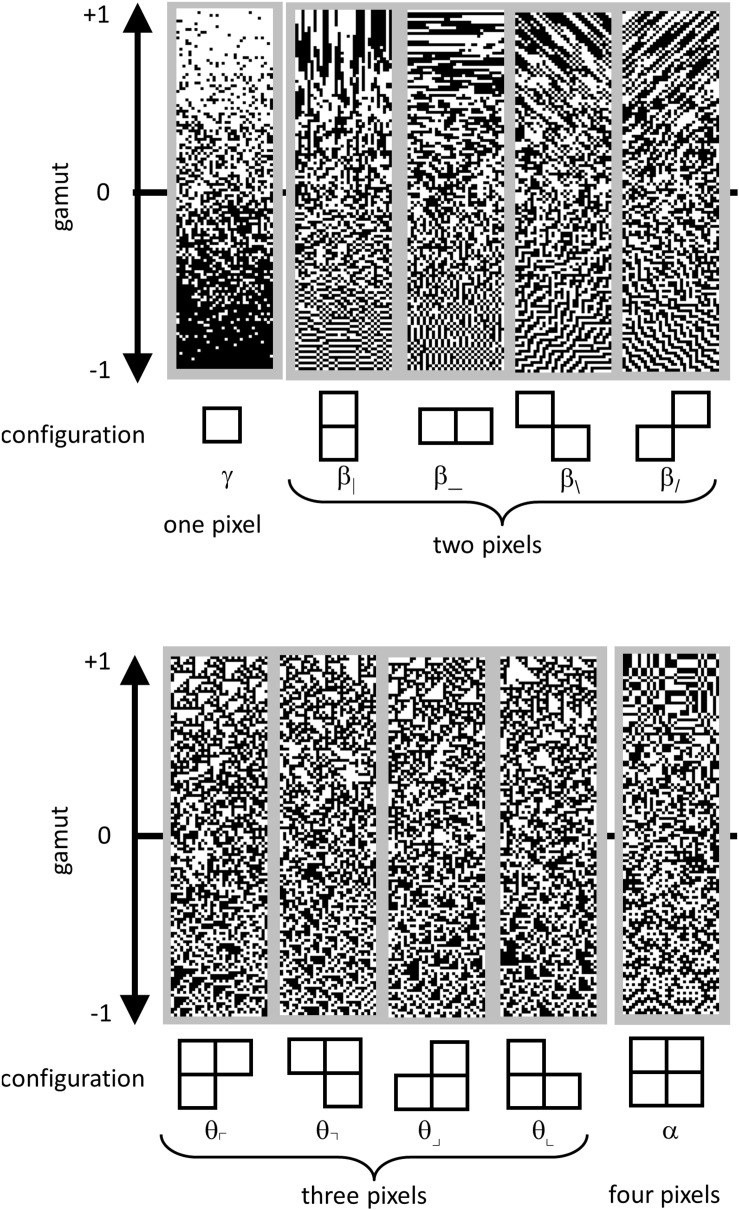
Parameterization of local image statistics. Each local statistic (γ, β_|_, β___, β_\_, and β_/_, θ_⌜_, θ_⌝_, θ_⌟_, θ_⌞_, and α ) describes a specific kind of correlation between one or more pixels that form a template within a 2 × 2 neighborhood. Values of each statistic range from −1 to +1, with 0 indicating randomness. The strip above each template shows this gamut. Statistics are defined as follows. The one-point statistic γ is the difference between the probability that a check is white, vs. black: +1 means all white, −1 means all black. The two-point statistics β_|_, β___, β_\_, and β_/_ specify the probability that two nearby pixels match, minus the probability that they do not match: +1 means that they always match; −1 means that they always mismatch. The three-point statistics θ_⌜_, θ_⌝_, θ_⌟_, and θ_⌞_ specify the difference between the fraction of L-shaped regions that contain an odd number of white pixels, and the fraction containing an even number: +1 produces an excess of white regions, while −1 produces an excess of black regions. The four-point statistic α specifies the difference between the probability that the number of white pixels in a 2 × 2 block is even, vs. odd: +1 means that 2 × 2 blocks always contain an even number of white pixels, −1 means that 2 × 2 blocks always contain an odd number of white pixels. Since the present analysis was carried out after binarization at the median, γ was always close to zero, and not analyzed; it is shown for completeness. Adapted from Figure 1 of Victor et al. (2015), with permission of the copyright holder, Elsevier B.V.

These co-occurrence statistics, each of which ranges from −1 to 1, may be summarized as follows (Figure 4). There are four two-point statistics, denoted β_|_, β_, β_\_, and β_/_. These describe the statistics of pairs of pixels, in directions corresponding to the subscript. For example, β_|_ is defined as the probability that two vertically adjacent checks match, minus the probability that they do not match. So $\beta_{|}\,\text{=}\,1$ means that all 2 × 1 (vertical) blocks are either both black or both white, while $\beta_{|}\,\text{=}-1$ means that all 2 × 1 pixel blocks contain mis-matching pixels. Similarly, β_ is the probability of matching vs. mismatching pixels in a 1 × 2 (horizontal) block. β_\_ and β_/_ describe the probability that pixels which share a common corner either match or mismatch. Together, β_|_ and β_ will be referred to as the cardinal β ’s; β_/_, and β_\_ will be referred to as the oblique β ’s.

There are four three-point statistics, denoted θ_⌜_, θ_⌝_, θ_⌟_, and θ_⌞_. These coordinates indicate the probability of that L-shaped regions containing an even or an odd number of white pixels. θ =1 means that there is always an odd number of white pixels in such a region, and θ = −1 means that there is always an even number of white pixels in the such a region, i.e., an odd number of black pixels. As shown in Figure 4, high or low θ values create regions with triangular shapes.

The four-point statistic α indicates the probability that the parity of the number of white pixels in a 2 × 2 block is even: α = 1 means that 2 × 2 blocks always contain an even number of white pixels and α = −1 means that 2 × 2 blocks always contain an odd number of white pixels.

For completeness, we mention that a complete parameterization of 2 × 2 block probabilities includes the one-point statistic, γ , which is defined as the probability of a white check minus the probability of a black check. However, since block probabilities were computed after binarization at the median, this statistic was forced to zero, and was not analyzed.

Thus, the nine quantities $\left\{\beta_{|},\beta \,\_,\beta_{\backslash },\text{ and }\,\beta_{/},\theta_{⌜},\theta_{⌝},\theta_{⌟},\theta_{⌞},\,\text{ and }\,\alpha \right\}$ are independent parameters that specify the probabilities of the 2 × 2 blocks. We determined these quantities for each ROI, and then examined their means, variances, and covariances across the ROI’s within each database. To determine confidence limits on these estimates, we used a bootstrap procedure (500 resamplings). Resamplings were done with replacement of each slice. Each time a slice was drawn, a new square ROI was randomly chosen from the maximal rectangle within its convex hull.

To enable a direct comparison to natural images, we accompany the analysis of MRI images by a parallel analysis of natural scenes, recomputed from the raw data of Hermundstad et al. (2014), which was provided by Ann Hermundstad. As in that study, natural images were only analyzed after twofold downsampling (*N*=2,*R*=32), to avoid possible camera-related artifacts.

## Results

We begin by describing the power spectra of each database of images. We then describe the two-, three-, and four-point correlations of the whitened binarized images, as these capture non-Gaussian aspects of image patches.

### Spectral Analysis

**FIGURE 5:**
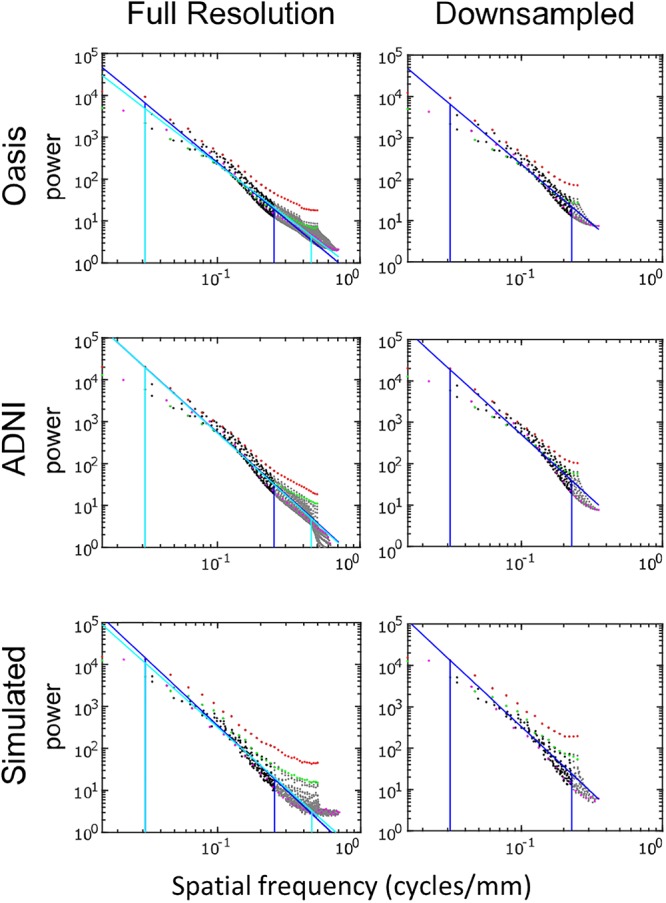
Power spectra of T1 MRI images, log-log plot. The simulated T1 images were constructed with 3% added noise; for further details, see text. Spectral estimates along the horizontal axis are shown in red, vertical in green, and oblique in magenta. The regression line and the frequency range used to determine the regression are shown for the full-resolution images in cyan and for the 2 × 2 downsampled images in blue (with the latter superimposed on the full-resolution analysis for comparison purposes).

Figure 5 shows the spatial power spectra of images in the T1-weighted databases, plotted as a function of the magnitude of spatial frequency, along with the corresponding quantities computed from simulated T1-weighted data. For each database, there is a broad range in which the power spectrum is approximately a power-law function of spatial frequency, i.e., $P\,\left(\vec{k}\right)\approx A\,{\left|\vec{k}\right|}^{-\lambda }$. The spectral slope (Table 2) is slightly shallower for the OASIS database (λ in the range 2.6–3.0) than for the ADNI database (3.0–3.2). Within each database, the spectral slope is similar for images analyzed at full resolution or after downsampling, and for horizontal vs. vertical vs. oblique spatial frequencies (Figure 5). Across these analyses, there is a flattening of the slope at the highest spatial frequencies, suggesting that noise in the imaging process, which would be expected to be approximately white (McVeigh et al., 1985), is a contributing factor in this range. Spectra computed from simulated T1-weighted images have a similar spectral slope (bottom row of Figure 5), and, as expected, the spectral slope for simulated images becomes progressively flatter as more noise is added (Table 2).

**TABLE 2:** Spectral slopes of brain MRI images and simulated images.

	**Full resolution**			**Downsampled**		
	**Slope**	**Confidence**	**Limits**	**Slope**	**Confidence**	**Limits**
**T1 images**						
OASIS	2.61	2.67	2.56	2.88	3.00	2.75
ADNI	3.09	3.15	3.03	3.11	3.24	2.97
Simulated, no noise	3.86	3.98	3.73	3.27	3.45	3.09
Simulated, 3% noise	3.03	3.11	2.94	3.18	3.36	3.01
Simulated, 5% noise	2.16	2.22	2.09	2.87	3.02	2.73
Simulated, 7% noise	1.86	1.92	1.81	2.70	2.82	2.58
**FLAIR images**						
TNS healthy	2.82	2.92	2.71	2.25	2.43	2.07
TNS patient	2.85	2.96	2.75	2.21	2.36	2.05
Simulated, no noise	3.64	3.74	3.55	2.56	2.67	2.46
Simulated, 3% noise	3.39	3.47	3.31	2.55	2.66	2.44
Simulated, 5% noise	3.12	3.18	3.06	2.53	2.63	2.42
Simulated, 7% noise	2.84	2.89	2.79	2.49	2.60	2.39

*Slopes were determined by regression of log(power) vs. log(spatial frequency) from 0.031 cycles/mm to 10% below the Nyquist frequency (0.45 cycles/mm for images processed at full resolution, 0.225 cycles/mm for the downsampled images); this range is shown by the vertical blue and cyan lines in Figures 5, 7. Confidence limits (95%) were determined via t statistics, as implemented by Matlab’s regress.m routine.*

**FIGURE 6:**
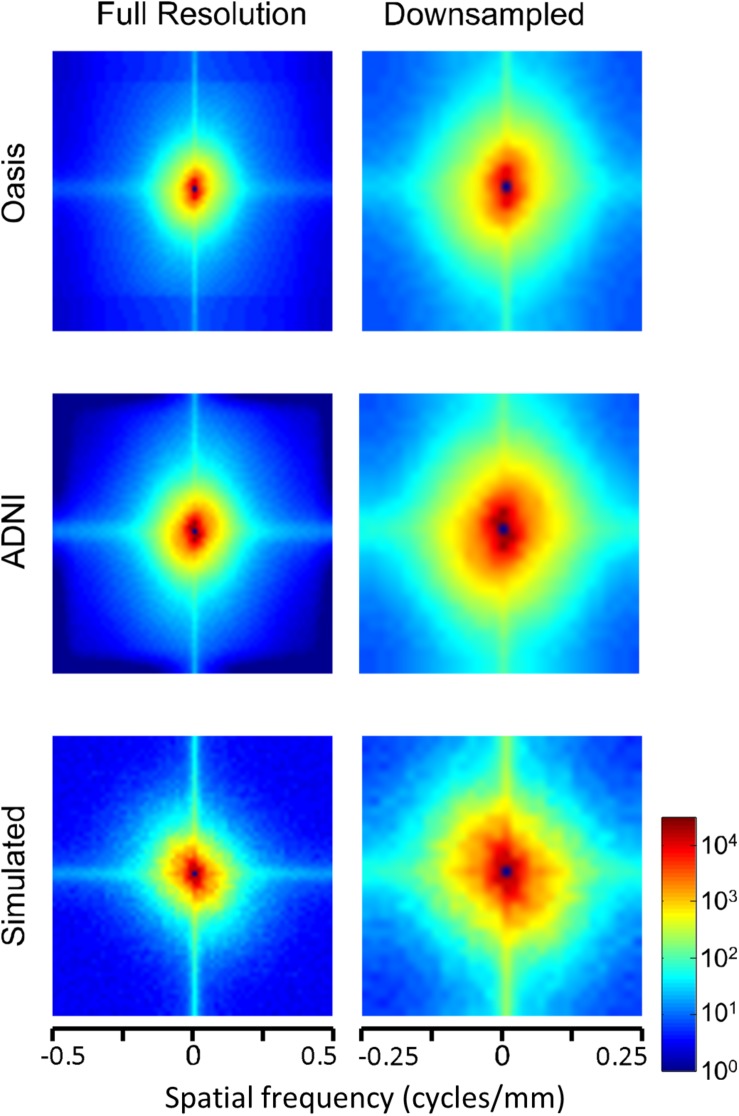
Power spectra of T1 MRI images (data of Figure 5), as a heatmap. Note that the color scale is logarithmic, and that the spatial frequency axes are expanded for the downsampled images (right column).

The two-dimensional power spectra (Figure 6) show that the MRI image spectra have moderate anisotropies. In both T1-weighted databases, there is a prominent excess of power along the horizontal and vertical axes, amounting to a 10-fold excess at the highest spatial frequencies. Away from the axes, the iso-intensity contours show a mild deviation from circularity, indicating a relative decrease of power in the oblique directions. Simulated T-weighted images share these features: they also have an excess of power along the horizontal and vertical axes, to a similar extent as the MRI images drawn from the database.

**FIGURE 7:**
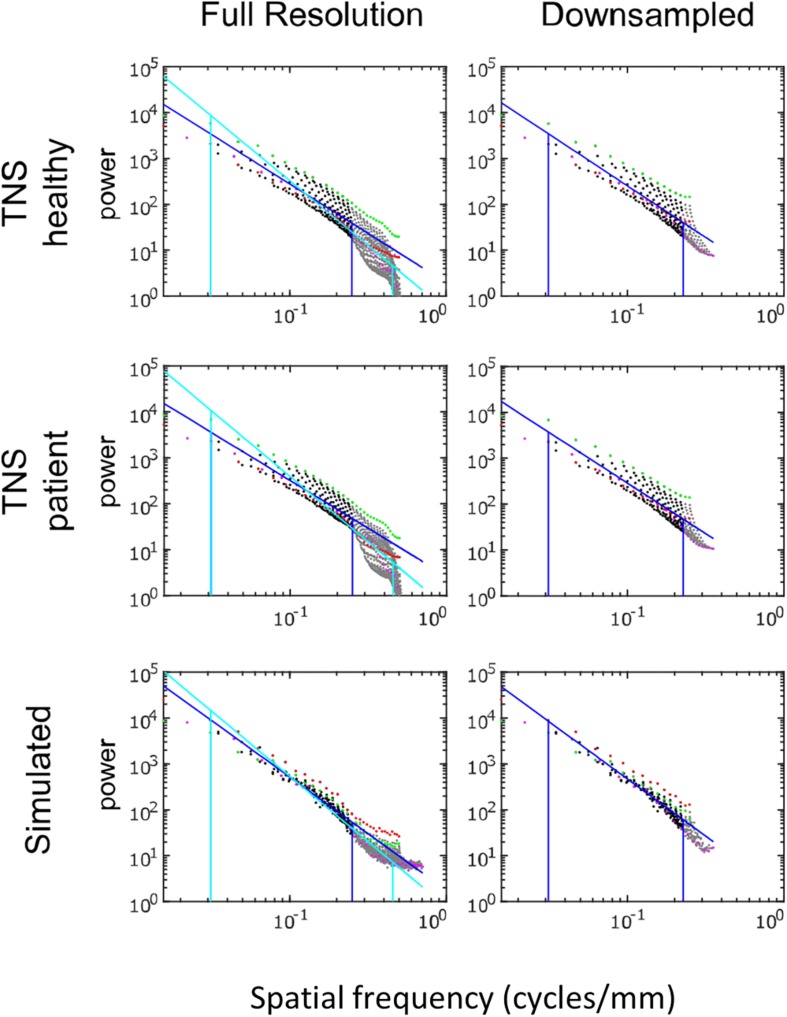
Power spectra of FLAIR MRI images, log–log plot. The simulated FLAIR images were constructed with 7% added noise; for further details, see text. Plotting conventions as in Figure 5.

**FIGURE 8:**
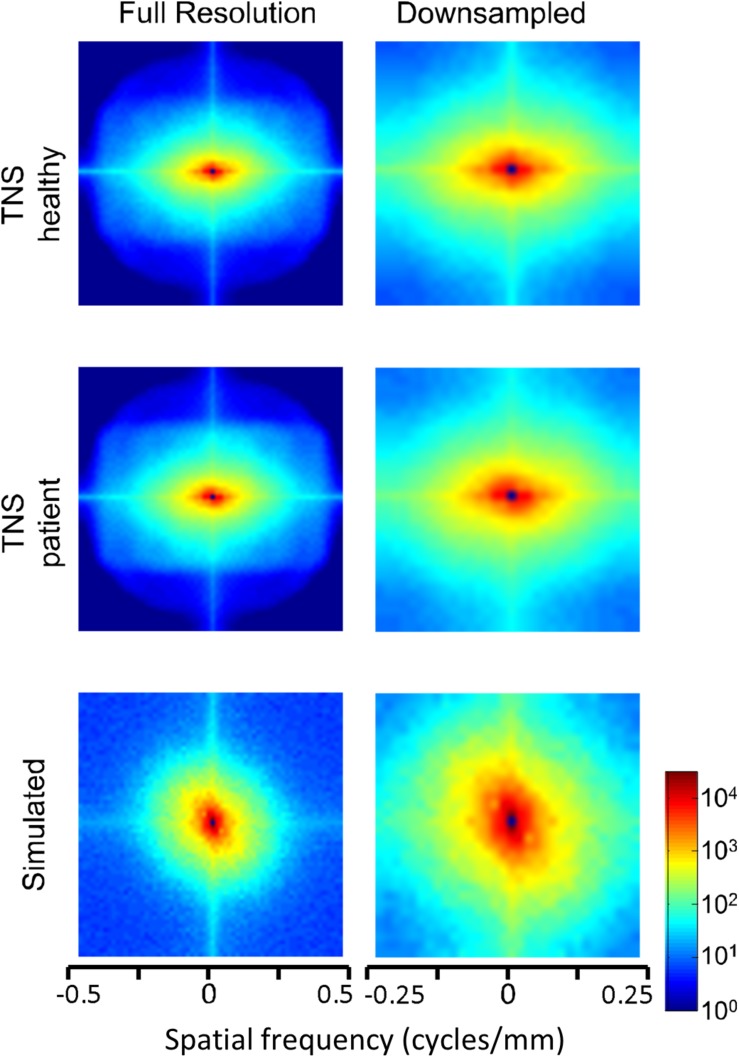
Power spectra of FLAIR MRI images (data of Figure 7), as a heatmap. Note that the color scale is logarithmic, and that the spatial frequency axes are expanded for the downsampled images (right column).

Power spectra of FLAIR images, taken from the TNS database, show similar characteristics (Figures 7, 8 and Table 2). The TNS database was subdivided into images from both healthy volunteers and patients; slopes were closely similar for the two subsets: 2.82 vs. 2.85 (healthy volunteers vs. patients) without downsampling, 2.25 vs. 2.21 with downsampling; overlapping confidence limits in both cases. Healthy volunteers and patients also both showed an excess of power, primarily along the vertical axis, of approximately a factor of three at the highest spatial frequencies. The spectral slope of the FLAIR images was reproduced by simulated FLAIR images with 7% noise added (lower row of Figures 7, 8). Simulated FLAIR images also showed anisotropy to a similar degree, but (in contrast to the database images) greater power along the horizontal axis than the vertical axis – suggesting that this anisotropy results from aspects of the imaging process that are not captured by the simulations.

In sum, across imaging modalities, MRI spectra had slopes in the range 2.2–3, a range that was consistent with simulated MRI images with modest amounts of noise added. There were moderate anisotropies as well, most of which were present in the spectra of the simulated images, indicating that most of the anisotropies are likely consequences of brain anatomy, rather than of image acquisition or reconstruction. Such anisotropies are not surprising, as subjects are positioned in the scanner in a stereotyped fashion, and brain anatomy at the MRI scale has numerous anisotropic structures, such as the corpus callosum. However, not all of the anisotropies of the FLAIR images were recapitulated by the simulated images, indicating that anisotropies due to imaging and reconstruction also play a role in the TNS datasets.

### Local Image Statistics

We now consider the local image statistics of MRI images. In brief (see section “Materials and Methods,” Figure 3, and Hermundstad et al., 2014), the approach consists of whitening the images followed by binarization, and then tabulating the configurations in 2 × 2 neighborhoods of pixels within each ROI. This tabulation is carried out in terms of a 9-parameter set of descriptors (Figure 4), which are grouped into co-occurrence statistics involving two points that share an edge (β_, β_|_), two points that share a corner (β_/_, and β_\_), three points in an L-shaped configuration (θ_⌜_,θ_⌝_, θ_⌟_, and θ_⌞_), and four points in a 2 × 2 neighborhood (α ). Note that this local statistical analysis is complementary to the characterization via power spectra, and focuses on non-Gaussian aspects of the images: for a Gaussian image ensemble, the whitening step would remove all correlations, and these nine parameters, which are the multipoint correlations of the binarized, whitened, images, would all be zero.

We consider the mean and standard deviations of these local statistics measured in each ROI. The mean values capture the average characteristics of all ROIs within a database, and thus focus on overall characteristics of each set of images. The standard deviations describe the characteristics that distinguish one ROI from another, and thus focus on characteristics that are useful for analyzing individual MRI images.

**FIGURE 9:**
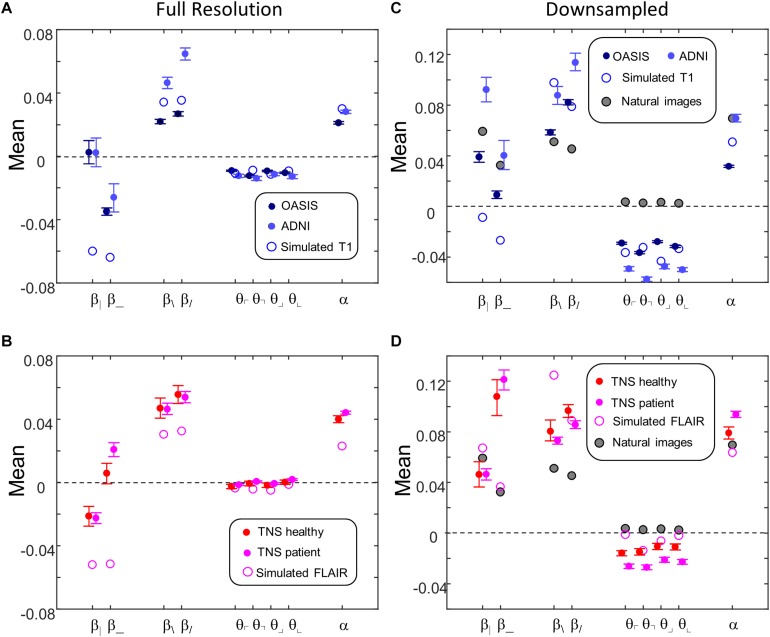
Means of local image statistics for real (solid symbols) and simulated (open symbol) MRI images. **(A,B)** Full resolution [(*R*,*N*)=(64,1)]; **(C,D)** after 2 × 2 downsampling [(*R*,*N*)=(32,2)]. In panels **(C,D)**, values for the natural image database of Hermundstad et al. (2014) are shown (shaded symbols). For MRI images, error bars indicate 95% confidence intervals for the mean across ROIs, computed via bootstrap. Symbols are jittered horizontally to avoid overlap.

Figure 9 shows the mean values of the image statistics for images analyzed at full resolution (Figures 9A,B), and after twofold downsampling (Figures 9C,D). At both scales, there were substantial differences in mean statistics obtained from the different databases. For example, at full resolution, vertical two-point correlations (β_|_) were zero or slightly positive for the T1 databases (Figure 9A) but negative for the FLAIR databases (Figure 9B), while horizontal two-point correlations (β_) were negative for the T1 databases (Figure 9A) but positive for the FLAIR databases (Figure 9B). Other differences were present for three- and four-point correlations at full resolution, and for many of the statistics after twofold downsampling (e.g., β_ and the three-point correlations θ in Figures 9C vs. 9D). In the downsampled analysis, there were also differences between the two T1 databases across all local image statistics (Figure 9C). For the simulated MRI images, many of the mean values differ from mean values obtained from actual MRI images, for both sequence types and at both scales.

This complex pattern of variations shows that the local statistical analysis is sensitive to non-Gaussian aspects of the MRI images, and validates that they can identify distinguishing features of the databases. Such variation in the mean values of the local statistics is not surprising, as these databases differ in the MRI intensity histogram, the physics of signal generation and, possibly, differences in the reconstruction process and instrument noise.

Figures 9C,D also shows the mean values of local image statistics obtained from natural images. Note that this analysis shows a different pattern: for example, three-point statistics for natural images have a mean near zero, rather than the negative values seen in all the MRI databases.

**FIGURE 10:**
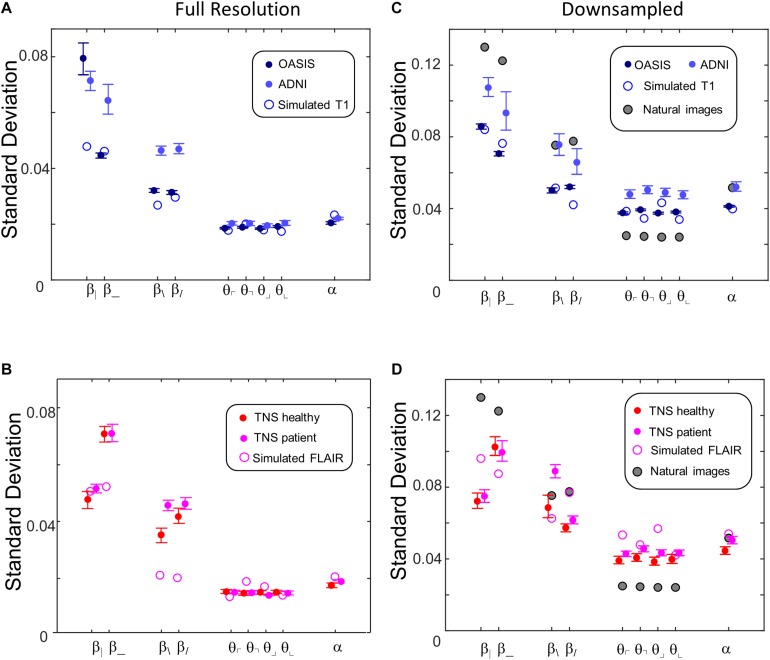
Standard deviations of local image statistics for real (solid symbols) and simulated (open symbol) MRI images. **(A,B)** Full resolution [(*R*,*N*)=(64,1)]; **(C,D)**: after 2 × 2 downsampling [(*R*,*N*)=(32,2)]. For MRI images, error bars indicate 95% confidence intervals for standard deviation across ROIs, computed via bootstrap. Other details as in Figure 9.

Figure 10 shows the standard deviations of these image statistics within each database, which assays the extent to which the image statistics serve to distinguish one ROI from another. In contrast to the behavior of the means of the statistics, the standard deviations show a simpler and more consistent pattern. Across all databases, two-point statistics in the vertical and horizontal directions (β_, β_|_) have the largest standard deviations, followed by two-point statistics in the oblique directions (β_/_, and β_\_). Three-point (θ ’s) and four-point (α ) statistics smaller but comparable standard deviations. This pattern is seen for images at full resolution (Figures 10A,B) and after 2 × 2 downsampling (Figures 10C,D), and also for the simulated T1 and FLAIR MRI images. The pattern is also consistent between images obtained from normal volunteers and patients within the TNS database.

While these characteristics are common to images from all the MRI databases and the simulated images, they differ from natural images (Figures 10C,D). This comparison is of interest because for natural images, the standard deviation of each parameter is closely correlated to human visual sensitivity for the corresponding feature (Figure 3 of Hermundstad et al., 2014), and this matching enables efficient processing of natural images. In comparison to natural images, horizontal and vertical two-point statistics of MRI images (β_|_ and β___ of Figures 10C,D) are less variable than in natural images. The opposite pattern is present for three-point statistics (θ ’s): standard deviations for the MRI statistics are above the corresponding values for natural images. These differences are consistent across real and simulated MRI images, and are substantially in excess of the confidence limits. That is, two-point correlations are relatively more stereotyped for MRI images than for natural images, while three-point correlations are relatively more stereotyped for natural images than for MRI. In other words, the relative importance of two- and three-point local image statistics for distinguishing among patches of MRI images differs from their relative importance in natural images.

The parametric descriptors of local image statistics shown in Figure 9 (mean) and Figure 10 (standard deviation) capture the main characteristics of their distributions in MRI images, and how they differ from the corresponding local image statistics of natural images. In contrast to the distribution of image statistics derived from filtering gray-level natural images, which typically have a substantial skewness (absolute value often greater than 1) and kurtosis (excess kurtosis often greater than 5) (Brady and Field, 2000; Lyu and Simoncelli, 2009; Hu and Victor, 2016), the skewnesses and kurtoses of the distribution of the local image statistics considered here are small: absolute value of skewness typically less than 0.3 and excess kurtosis typically less than 0.5 at the two resolutions. These are comparable to the values obtained from a parallel analysis of the natural image database. Additionally, of the 72 image statistics examined (nine kinds of statistics, four databases, two resolutions), all but one had a unimodal distribution; the exception (β___ in the OASIS database at full resolution) had a minor second mode accounting for approximately 20% of the mass. Finally, the covariances of the image statistics in the four MRI databases (not shown) are also similar to those of the corresponding statistics of natural images.

## Discussion

Here we present an analysis the image statistics of anatomical T1-weighted and FLAIR brain MRI images, focusing on specific aspects of non-Gaussianity – multipoint spatial correlations – for which human visual sensitivity is well-characterized (Hermundstad et al., 2014; Victor et al., 2015). The basic motivation for this focus is that fundamental image features such as lines and edges correspond to local high-order correlations, rather than to spectral characteristics (Oppenheim and Lim, 1981), and such high-order correlations are also critical to visual detection of these features (Morrone and Burr, 1988).

As in prior work characterizing natural scenes (Tkacik et al., 2010; Hermundstad et al., 2014), these aspects of non-Gaussianity are quantified by multipoint correlations, computed from ROIs that have been subjected to spectral whitening. Consequently, the statistical characterization of an image set via local multipoint correlations is complementary to characterization of its global statistics via the power spectrum. Thus, the spatial power spectra of brain MRI’s are of interest in their own right, not only as a preliminary step in determining the multipoint image statistics. Perhaps surprisingly, previous studies of the spatial power spectrum of brain MRI images do not appear to be available [an extensive search revealed only Ramon et al. (2009), which considered the power spectrum of the cortical volume as a whole, and work such as McVeigh et al. (1985), which examined the power spectrum of imaging noise]. We first discuss these power spectra, and then the multipoint correlations.

The power spectra of T1-weighted and FLAIR MRI images, along with simulations, have spectral slopes that are in the range 2.2–3 (Figures 5–8 and Table 2). Similar values (2.8–3) have been reported for digital breast mammograms (Burgess et al., 2001; Abbey et al., 2012). However, this apparent similarity does not take into account the expected differences between imaging processes that involves projection, such as mammography, and those that involve section, such as tomography. As Metheany et al. (2008) showed, under the assumption that a power law describes anatomical correlations, slopes are expected to be one unit lower for tomographic sections than for projections, i.e., $\lambda_{\mathrm{section}}=\lambda_{\mathrm{proj}}-1$. Consistent with this prediction, breast computed tomograms have a shallower spectral slope (∼1.86, Metheany et al., 2008) than digital breast mammograms. The slopes reported for breast computed tomograms are substantially shallower than the slopes that we find for the sectioned images of brain MRI.

The spectral slopes of MRI images also differ substantially from that of images of natural scenes, which is well-known to be 1.8 to 2.2 (Field, 1987; Ruderman and Bialek, 1994; Ruderman, 1997; Torralba and Oliva, 2003). As in radiographs, image formation in natural scenes involves projection. However, the effect of projection vs. section on spectral slope, determined in Metheany et al. (2008), cannot be rigorously applied to natural images: the analysis of Metheany et al. (2008) treats projection as linear superposition, but in contrast to objects in radiographs, objects in natural scenes are largely opaque. Nevertheless, it is unlikely that the difference in spectral slope between images of natural scenes and MRI sections is due to opacity vs. transparency, as Metheany et al. (2008) predicts that tomographic slices should have a shallower, rather than a steeper, slope. Moreover, Zylberberg et al. (2012) found that transparency vs. opacity has little effect on the spectral slope. Rather, in the context of a “dead leaves” model, the main determinant of spectral slope is the object size distribution (Ruderman, 1997). But while opacity and occlusion may not be important for determining spectral slope, they are likely critical for other key aspects of natural images (Zoran and Weiss, 2012), such as T-junctions, which are manifest in local image statistics (see below).

The analysis of local image co-occurrence statistics reveals substantial non-Gaussian spatial characteristics of MRI images (Figures 9, 10). These differences are independent of the spectral slope of the original images, as the image statistics are calculated after spectral whitening. The mean values of these statistics (Figure 9) depend on the imaging sequence, which is not surprising given that each MRI sequence has its own tradeoffs between bandwidth, noise, contrast and artifacts, and, *a priori*, any of these could have had a role in determining the statistics of resulting images.

While these mean values reflect the typical values of image statistics and therefore characterize each database as a whole, their standard deviations across ROIs (Figure 10) reflect the features that distinguish one ROI from another, and therefore (along with many other features) that may be useful for making diagnostic distinctions. The standard deviations of the statistics vary somewhat across sequence types (T1-weighted and pre-contrast FLAIR), but there are prominent commonalities. Variability of two-point statistics in horizontal and vertical directions was greatest; variability of two-point statistics in oblique directions was less, and variability of three- and four-point statistics were comparable to each other and less than the variability of the two-point statistics.

Based on this consistency across three databases representing two sequence types as well as simulated structural brain MRI images, we infer that the statistical characteristics presented here are not due to the physics specific to each kind of MRI sequence, or filtering and artifacts that might arise in the process of data acquisition or reconstruction. Rather, they appear to reflect the geometry of the underlying brain tissue at the resolution of MRI.

Figures 10C,D shows a consistent difference between the multipoint statistics of MRI images and those of natural scenes: three-point statistics are substantially more variable in the former than in the latter. The basis for this difference is unclear, but factors related both to material properties (“stuff” Adelson, 2001) and shape may contribute. With regard to material properties, the three-point statistic is, explicitly, a texture statistic (Victor and Conte, 2012; Victor et al., 2015), so differences between three-point statistics may arise from differences in material properties. With regard to shape, three-point statistics reflect, at least in part, the characteristics of T-junctions. This is because the binarization step of the image-processing pipeline transforms T-junctions into configurations of checks based on the relative intensities of the three regions that meet at a point. While T-junctions are present in both MRI images and images of natural scenes, they arise by different mechanisms. For sections of a 3D volume of space-filling structures – such as brain MRI – T-junctions arise whenever the section passes through loci where three structures meet. In images of natural scenes generated by projection, however, T-junctions arise when the boundary between two objects is partially occluded by a third object that is closer. MRI images, being tomographic, would be expected to have very few T-junctions generated by occlusion.

### Analysis Scale

Natural scenes are often considered to be scale-invariant, or at least approximately so. Approximate scale invariance is the expected consequence of images that are formed by random occlusive objects with a wide range of sizes, viewed from a wide range of distances (Field, 1987; Ruderman and Bialek, 1994; Ruderman, 1997). However, there is no corresponding expectation of scale-invariance for brain MRI: human brains are quite similar in size, and MRI images of them are typically viewed only at a narrow range of distances. Thus, while it is likely that brain MRI image statistics will vary according to analysis scale, there is a restricted range of scales at which they are viewed. For example, a same-size brain image acquired and displayed with 1 mm resolution, viewed at 57 cm, provides pixels that are 0.1°, i.e., 6 arc-min. Viewing a smaller image will decrease the angular subtense of a pixel; enlarging the image or decreasing the viewing distance will increase it – but not typically by more than a factor of two. Thus, the typical resolution with which brain MRIs are viewed is approximately 3–12 arc-min per pixel, similar to the range in which human sensitivity to local image statistics is approximately scale-invariant [2.8 to 14 arc-min per pixel (Victor et al., 2015)], and corresponding to spatial frequencies (2.5–10 cycles per degree) for which human contrast sensitivity is highest (Campbell and Robson, 1968). This is the range we focused on.

As described above, we found a consistent difference between local image statistics at this scale, and a parallel analysis of natural images (Hermundstad et al., 2014). In the direct comparison, analysis parameters were (*R*,*N*)=(32,2), i.e., local images statistics were calculated from square regions of 32 × 32 blocks, each of which was obtained by 2 × 2 downsampling of the original image pixels. The natural image analysis did not use the full image resolution, both out of concern for camera artifacts and to ensure that the blocks used in the analysis were within the range of human resolution (1.3 arc-min per pixel with 2 × 2 downsampling).

This matching of image-processing parameters necessarily leads to a mismatch between the angular resolution of the MRI and the natural-image patches from which local image statistics are determined, since the original images have quite different native resolutions. However, it is unlikely that the resolution difference accounts for our finding that third-order local image statistics carry a disproportionate amount of information in brain MRI. As Figures 10A,B show, doubling the resolution of the MRI analysis to the range of 3 arc-min as typically viewed, by taking *N* = 1 (no downsampling), does not change the finding that third-order statistics almost as variable as fourth-order statistics. Conversely, as shown in Figure 3 of Hermundstad et al. (2014), halving the resolution of the natural-image analysis to 2.6 arc-min per pixel, by taking *N* = 4, leaves the relationship between the informativeness of third-order statistics, on the one hand, and second- and fourth-order statistics, on the other, substantially unchanged.

### Potential Implications

Above, we considered possibilities for the origin of the distinctive aspects of three-point statistics in brain MRI images. We now turn to speculation about their relevance for image interpretation: understanding human expertise, and possible implications for machine vision algorithms.

With regard to expertise, we first note that the relative un-importance of three-point statistics in natural scenes is mirrored by a relative lack of visual sensitivity to them. That is, in natural scenes, four-point local statistics are more informative than three-point ones, and, correspondingly, typical human observers are more sensitive to textures defined by four-point correlations than to textures defined by three-point correlations (Hermundstad et al., 2014; Victor et al., 2015). This finding has been interpreted (Hermundstad et al., 2014) as a form of efficient coding (Barlow, 1961): an evolutionary (or possibly developmental) adaptation in which a sensory system’s limited computational resources are allocated to process the most critical aspects of sensory stimuli. The matching of typical human visual sensitivity to the informative statistics of natural images necessarily implies a mismatch, albeit a subtle one, to the statistics that characterize MRI images.

Clearly the mismatch is not so severe as to prevent effective interpretation of these images, but its existence raises the possibility that development of expertise during clinical training – along with cognitive factors – may also have a component due to, or be accompanied by, an increase in clinicians’ sensitivities to these low-level visual features. Additionally, there are modest inter-individual differences in sensitivity to local image statistics (Victor and Conte, 2012; Victor et al., 2015); raising the possibility that individuals with relatively greater sensitivity to the third-order statistics that are informative in brain MRI images will more readily develop neuroradiographic expertise. Further work is needed to determine the significance of these factors.

The subtle mismatch between brain MRI image statistics and those of natural scenes also raises the possibility of developing image-processing algorithms to modify standard MRI images so that their statistics are closer to those of natural images. We mention this only as a speculation, recognizing that the considerable challenges of this approach include the need for experts to become accustomed to altered images.

With regard to machine vision applications, a common strategy is to use local image statistics as features that are the inputs to a machine-learning algorithm. For example, one recent study demonstrated the utility of second-order statistics in distinguishing T2 MRI images of normals and patients with Alzheimer’s disease (Aggarwal and Agrawal, 2012); a second demonstrated superiority of machine analysis of MRI images to human analysis in distinguishing brain tumor from radionecrosis (Tiwari et al., 2016). While the use of image statistics to capture texture is prevalent, explicit use of three-point statistics – which could be extracted by application of a non-linearity with substantial odd-order components to local neighborhoods in the image in an otherwise-standard CNN architecture – appears to have been overlooked. The present study suggests that these statistics may be important for brain MRI images. However, diagnostic utility of a set of texture features depends not only on their variability across ROIs – the present focus – but also on the extent to which they can discriminate images from normal and diseased brains.

## Data Availability

The datasets generated for this study are available on request to the corresponding author.

## Author Contributions

YX and JV designed the study and wrote the initial manuscript. YX carried out the analysis. All authors edited the manuscript, contributed intellectually to the work, and approved it for publication.

## Conflict of Interest Statement

The authors declare that the research was conducted in the absence of any commercial or financial relationships that could be construed as a potential conflict of interest.
